# Supplementary material for: The origin recognition complex requires chromatin tethering by a hypervariable intrinsically disordered region that is functionally conserved from sponge to man
Source: Nucleic Acids Res. 2024 Feb 21;52(8):4344–60. doi: 10.1093/nar/gkae122 (PMC11077064; doi:10.1093/nar/gkae122)
Supplement: gkae122_supplemental_file [file gkae122_supplemental_file.pdf]

Supplemental Figure S1

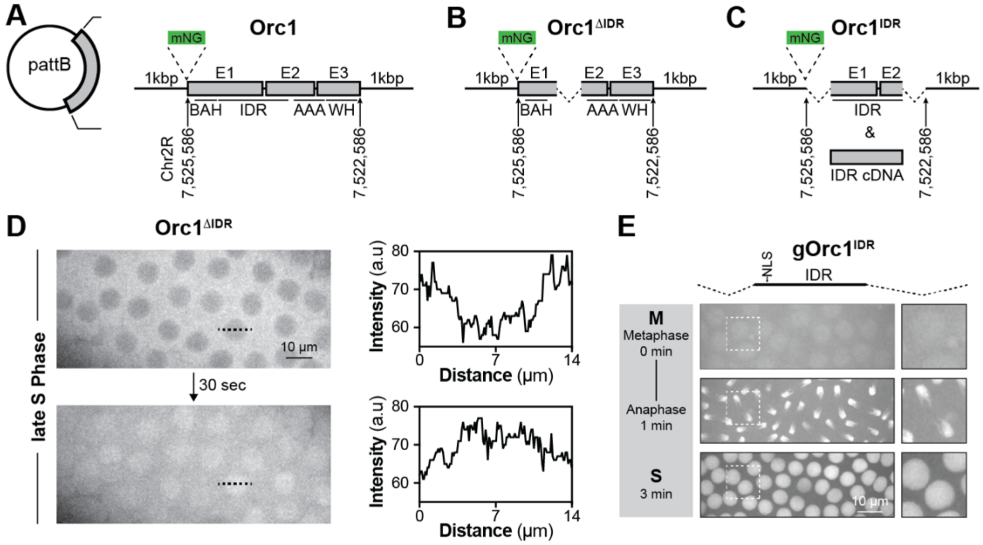

Supplemental Figure S1: The fly Orc1 IDR is necessary and sufficient for chromatin recruitment *in vivo*. A-C) Graphical representation of Orc1 transgenes generated in vector pattB, including full-length Orc1 (Orc1, A), an IDR deletion construct (Orc1$^{\Delta IDR}$, B), and the IDR alone (Orc1^{IDR}, C). D) Imaging in embryos in late S-phase reveals a dramatic change in Orc1$^{\Delta IDR}$ nuclear localization. Included are line intensity profiles of mNeonGreen-Orc1$^{\Delta IDR}$ signal. E) Chromatin localization of an Orc1^{IDR} transgene produced from genomic DNA.

Supplemental Figure S2

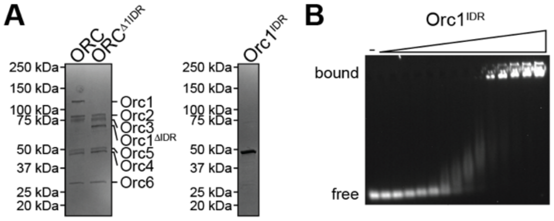

Supplemental Figure S2: The Orc1 IDR facilitates ATP-independent DNA binding *in vitro*. A) SDS-PAGE of purified *Dm*ORC holocomplex (ORC) and an Orc1 IDR deletion construct (ORC$^{\Delta 1IDR}$). Second gel is SDS-PAGE of purified *Dm*Orc1^{IDR}. B) EMSA analysis of *Dm*Orc1^{IDR} DNA-binding. Each lane represents a 2-fold dilution of Orc1 (0.6 – 5,000 nM) which was combined with 2 nM Cy5-dsDNA in Assay Buffer (50 mM HEPES pH 7.5, 150 mM KGlutamate, 10% glycerol, 1 mM BME).

Supplemental Figure S3

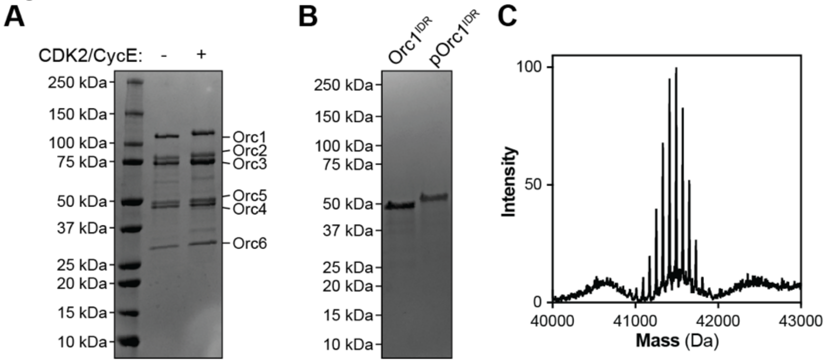

Supplemental Figure S3: Phosphorylation of the Orc1 IDR regulates DNA and chromatin binding. A) SDS-PAGE analysis of non-phosphorylated ("-") and CDK2/CycE phosphorylated ORC ("+"). B) SDS-PAGE analysis of purified $Orc1^{IDR}$ and CDK2/CycE phosphorylated $Orc1^{IDR}$ ($pOrc1^{IDR}$). C) Intact mass spectrometry reveals that $pOrc1^{IDR}$ has been phosphorylated approximately 15 times.

Supplemental Figure S4

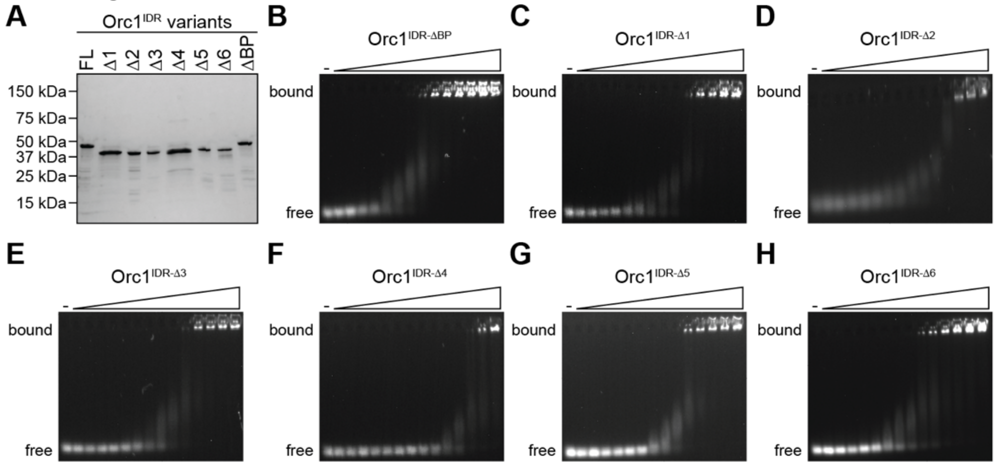

Supplemental Figure S4: The Orc1 disordered region possesses multiple redundant DNA binding motifs. A) SDS-PAGE analysis of purified *Drosophila* Orc1^{IDR} and the listed deletion constructs. See MATERIALS AND METHODS for indices of deleted amino acids. B-H) EMSA analysis of Orc1^{IDR} deletion construct binding to Cy5-dsDNA.

Supplemental Figure S5

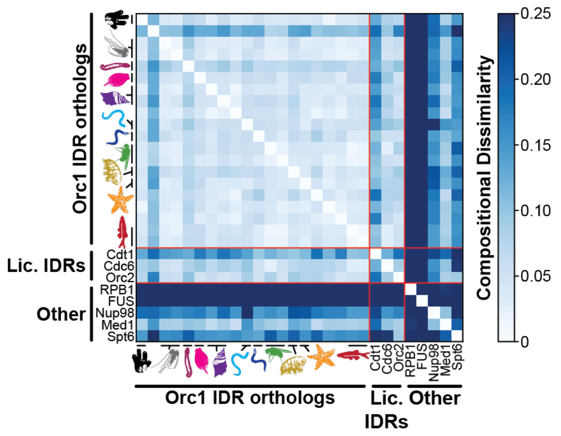

Supplemental Figure S5: Metazoan Orc1 IDRs are compositionally distinct from other IDR functional classes. Pairwise comparison of compositional dissimilarity in metazoan Orc1 IDRs ("Orc1 IDR orthologs"), other licensing factor IDRs ("Lic. IDRS", including human Cdt1 residues 1-167, Cdc6 residues 1-140, and Orc2 residues 1-237), and IDRs from other functional categories ("Other", including human Rpb1 residues 1485-1970, Med1 residues 538-1581, Spt6 residues 1-310, Nup98 residues 1-728, and FUS residues 1-287). Lighter colors indicate sequences with similar sequence composition.

Supplemental Figure S6

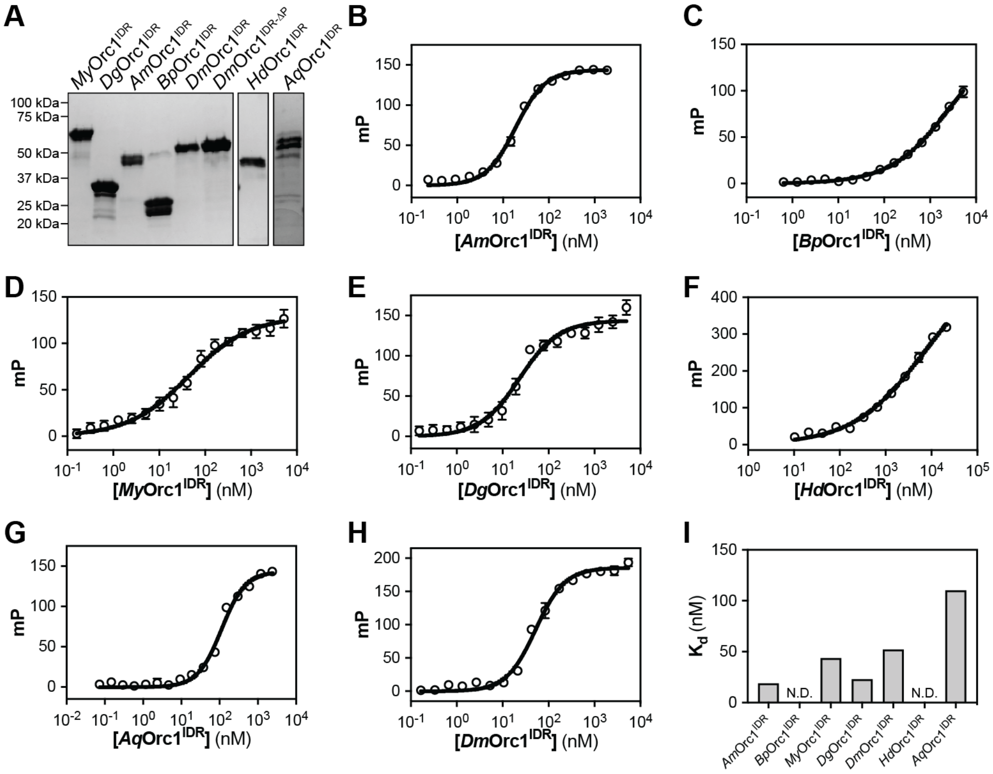

Supplemental Figure S6: The Orc1 IDR is functionally conserved across metazoans. (A) SDS-PAGE of purified metazoan Orc1 IDRs (Mollusc (*My*), Annelid (*Dg*), Cnidaria (*Am*), Rotifer (*Bp*), Arthropod (*Dm* and *Dm*Orc1$^{IDR\text{-}\Delta P}$), Tardigrade (Hd), and Porifera (Aq)). (B-H) Fluorescence polarization DNA binding assays to assess the affinity of Orc1 IDR orthologs for FITC-dsDNA. The ortholog is indicated by the x-axis label. From these data the DNA-binding affinity (K_d) was calculated and (I) plotted for comparison. *Bp*Orc1^{IDR} (C) and *Hd*Orc1^{IDR} (F) binding curves did not reach saturation and therefore their K_d's were not determined (N.D.).

Supplemental Figure S7

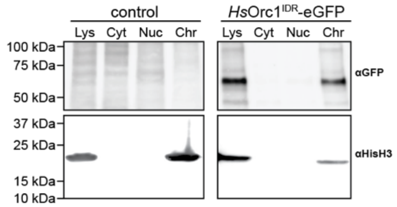

Supplemental Figure S7: HeLa cell fractionation to assess chromatin binding of *Hs*Orc1^{IDR}. HeLa cells transiently transfected with either an empty 6D vector ("control") or the 6D-*Hs*Orc1^{IDR} vector (for expression of GFP-tagged Orc1^{IDR}) were fractionated into cytosolic (Cyt), nuclear (Nuc), and chromatin-bound (Chr) fractions and analyzed by SDS-PAGE and western blotting. The initial lysate (Lys) was included as starting material. An anti-GFP (αGFP) antibody tracked fractionation of *Hs*Orc1^{IDR}-eGFP and an anti-Histone H3 (αHisH3) antibody was used to confirm successful fractionation of chromatin-bound proteins. The expected molecular weight of *Hs*Orc1^{IDR}-eGFP is 62 kDa.
